# The Knowledge, Attitude, Practices, and Satisfaction of Non-Invasive Prenatal Testing among Chinese Pregnant Women under Different Payment Schemes: A Comparative Study

**DOI:** 10.3390/ijerph17197187

**Published:** 2020-09-30

**Authors:** Wenjun Zhu, XiaoXiao Ling, Wenru Shang, Jiayan Huang

**Affiliations:** 1 School of Public Health, Fudan University, Shanghai 200433, China; 18211020046@fudan.edu.cn (W.Z.); 18111020026@fudan.edu.cn (W.S.); 2 Key Lab of Health Technology Assessment, National Health Commission of the People’s Republic of China, Shanghai 200433, China; 3 Department of Statistical Science, University College London, London WC1E 6BT, UK; rejbxli@ucl.ac.uk

**Keywords:** non-invasive prenatal testing, pregnant women, knowledge, attitude, practice, satisfaction, payment scheme, acceptability

## Abstract

Non-invasive prenatal testing (NIPT) for aneuploidy screening has been widely applied across China, and costs can affect Chinese pregnant women’s choices. This study aims to assess the knowledge, attitude, practices (KAP) and satisfaction regarding NIPT among pregnant women in China, and to further explore the relationship between payment schemes and women’s acceptability of and satisfaction with NIPT. A questionnaire survey was performed in Shenzhen and Zhengzhou, China, which separately applied “insurance coverage” and “out-of-pocket” payment scheme for NIPT. The major differences between the two cities were compared using chi-square test, Wilcoxon rank sum test, and propensity score matched analysis. Logistic regression models were applied to explore predictors for women’s acceptability and satisfaction. Compared with Zhengzhou participants, a higher proportion of Shenzhen women had heard of NIPT (87.30% vs. 64.03%), were willing to receive NIPT (91.80% vs. 80.43%) and had taken NIPT (83.12% vs. 54.54%), while their satisfaction level was lower. Having NIPT-related knowledge was associated with higher acceptability, and receiving genetic counseling helped to improve satisfaction. Besides, women with higher annual household incomes were more likely to take and be satisfied with NIPT. In conclusion, more attention should be paid to health education, subsidies for NIPT, and genetic counseling.

## 1. Introduction

Down syndrome (DS), which results from the dosage imbalance of genes located on human chromosome 21, is the most common form of inherited intellectual disability [1]. Moreover, there is a significantly higher risk of hearing loss, congenital heart defects, intestinal atresia, seizures, and childhood leukemia among DS patients [2,3]. According to the National Health Commission, there are 23,000–25,000 infants born with DS each year in China. From a societal perspective, the total lifetime economic burden of annual-born patients exceeds USD 1.4 billion in China [4]. Besides, China has not fully established the life-cycle social support network, rehabilitation system, and special education system for patients with intellectual disabilities [5,6]. Thus, the families of DS patients undertake most of the care responsibilities and this may lead to catastrophic health expenditure.

Prenatal screening is a way to evaluate pregnant women’s risk of carrying a fetus with DS. Commonly, two types of screening are available. One is the maternal serum screening (MSS), including first-trimester and second-trimester serum screening. For the former, a combination of nuchal translucency measurement, human chorionic gonadotropin (hCG) or serum free β-hCG, and pregnancy-associated plasma protein-A analyte levels measurement is provided [2]. For the latter, triple screen (hCG, alpha fetoprotein (AFP), and unconjugated estriol (uE3)) and quadruple screen (hCG, AFP, uE3, dimeric inhibin-A (DIA)) are available [7]. Besides, the integrated or sequential screening which combines first- and second-trimester screening can provide a higher detection rate than single screening [2]. The sensitivity and specificity of these screening methods vary from 0.77–0.92 and 0.93–0.96 respectively [8,9,10]. The other is the non-invasive prenatal testing (NIPT), which uses massively parallel sequencing of cell-free DNA in maternal plasma [11]. Compared with MSS, NIPT shows higher sensitivity and specificity of 99.7% and 100% [12]. In 2013, the National Society of Genetic Counselors (NSGC) and the American College of Medical Genetics and Genomics (ACMG) published statements and supported NIPT to be a reliable screening tool for DS, especially in high-risk pregnancies [13,14]. In 2015, the International Society for Prenatal Diagnosis (ISPD) stated that emerging evidence suggested that women with average risk could also benefit from NIPT [15]. These statements promote the application of NIPT around the world.

With the wide spread of NIPT, several studies have explored the knowledge, attitude, practices (KAP), and satisfaction of NIPT in different populations. Previous research has found that a majority of pregnant women (>60%) had heard of NIPT in Japan and Australia, but fewer women realized that, if a positive result was returned, then an invasive diagnosis was required [16,17]. Pregnant women’s preference for NIPT varied from 74% in Sweden [18] to 81% in the Netherlands [19]. For women who held a positive attitude towards NIPT, the most common reasons stated were “to assure the body’s health status” and “having a higher risk of carrying DS”; while for those who declined NIPT, the most common reasons were “opposed to terminating a pregnancy” and “sufficiently reassured by the MSS results” [19,20]. Except for the awareness of NIPT, researchers also found that costs could largely affect pregnant women’s attitudes and choices towards NIPT. A Swedish study found that the willingness-to-pay of pregnant women for NIPT was much lower than its current commercial price, and it might impose a negative effect on the practice of the test [18]. In terms of satisfaction, a study conducted by van Schendel et al. demonstrated that 97.5% of pregnant women did not regret receiving NIPT, one-third of whom would have even preferred to receive it earlier [21].

In 2016, the National Health Commission of China published the clinical guideline for NIPT practice. It is recommended that NIPT be offered at 12 through 22 weeks of gestation. The number of pregnant women, especially women with advanced maternal age, has been increasing, due to the implementation of the universal two-child policy in the same year [22]. It also increases the pressure of prenatal screening and the need for NIPT. Currently, the price of NIPT is about USD 100–250 in China, and the cost is an important influential factor for the use of NIPT in the Chinese population [23,24]. A previous study noted that comparing medical costs with the number of DS averted, the contingent screening strategy was a cost-effectiveness option. This strategy suggested women with high-risk MSS results to implement NIPT as a former step of invasive diagnosis [25]. Thus, from a societal perspective, the implementation of NIPT could be an economic option. Currently, the payment schemes of NIPT in China can be categorized into two main types: the “insurance coverage” scheme and the “out-of-pocket” scheme, as whether to cover NIPT into the social medical insurance list is decided by provincial bureaus. Under the former scheme, the personal expenses of NIPT would be certainly lower than the other. Thus, by investigating the KAP and satisfaction of NIPT among pregnant women in China, the present study aims to understand the influence of payment schemes on the acceptability and satisfaction towards NIPT, and to provide some advice for NIPT promotion.

## 2. Materials and Methods

### 2.1. Study Settings

A cross-sectional study on NIPT-related KAP and satisfaction among pregnant women was conducted in Shenzhen, Guangdong Province, and Zhengzhou, Henan Province, China. Guangdong Province belongs to the eastern region of China, and Shenzhen is one of its core cities. In 2018, the Gross Domestic Product (GDP) per capita of Shenzhen was USD 28,646.9, which was almost three times higher than GDP per capita of China (USD 9768.8). Henan Province is located in the central region of China, and Zhengzhou is its capital city. In 2018, the GDP per capita of Zhengzhou was USD 15,315.5, which was a little higher than the average level of China. In terms of geographic and economic considerations, Shenzhen represents the high-income eastern region of China, while Zhengzhou belongs to the middle-income central region of the country. Besides, NIPT is covered by social medical insurance in Shenzhen but not in Zhengzhou. In Shenzhen, the price of NIPT is USD 123.2, and 64.71% of the cost can be reimbursed by social medical insurance. While in Zhengzhou, NIPT costs USD 226.1, and all should be paid by pregnant women themselves, unless they purchase commercial health insurance. The two cities can represent the “insurance coverage” and “out-of-pocket” payment schemes respectively.

Because of the technological requirement, the majority of the NIPT services are provided by tertiary hospitals in China. A large tertiary Maternity and Child Health Hospital was selected as the survey site in each city. For Hospital A in Shenzhen, it advises pregnant women of all ages to take MSS first, and then take NIPT or invasive prenatal diagnosis based on its results. Hospital B in Zhengzhou recommends a similar strategy for women aged under 35, but it suggests that women aged over 35 should receive NIPT or invasive prenatal diagnosis directly, because of their higher risk of carrying a DS fetus. Both hospitals serve as the prenatal screening and diagnosis centers in the local region, and a broad variety of pregnant women can choose their service. In 2018, 26,640 and 40,231 pregnant women registered in Hospital A and B respectively, which were equivalent to 25.2% and 32.2% of the population born in the same year locally.

### 2.2. Recruitment of Participants

Pregnant women who have finished prenatal aneuploidy screening were recruited from January 2019 to September 2019. Assuming that the acceptance rate of NIPT among pregnant women was 70% [25], and to detect a rate difference of 10% between Shenzhen and Zhengzhou participants, 278 and 556 participants were required for two groups, to achieve 90% power and 2-sided type I error of 5% when using a simple random sampling method [26]. However, as it was difficult to perform simple random sampling in an outpatient survey, convenience sampling was applied instead, and we expanded the sample size to 600 and 1200 women in the two cities, to increase the representativeness. The recruitment took place in the waiting room, where women can take their prenatal screening result reports from automated printing machines and make an appointment with the doctor. Nurses would explain the summary of research to pregnant women and invite them to complete a questionnaire about NIPT. Written informed consent was obtained from each participant. The study was conducted in accordance with the Declaration of Helsinki, and it was approved by the Medical Research Ethics Committee, School of Public Health, Fudan University (IRB00002408 and FWA00002399).

### 2.3. Data Collection

An anonymous questionnaire was developed based on the characteristics of NIPT and the prenatal screening guidelines [2]. After the first draft was finished, a focus group discussion was conducted to select and revise the items of the questionnaire. The discussion convened 2 experts in NIPT, 2 experts in payment and medical insurance, and 2 experts in KAP. After that, the revised questionnaire was reexamined by a pilot study. We investigated 100 pregnant women in each survey site, to test whether the women could understand the meanings of each item.

In all, the questionnaire had three components. The first part included the socio-demographic characteristics of pregnant women, regarding age, residence, educational level, occupation, parity, number of family members, annual household income, and types of medical insurance purchased. The second part evaluated NIPT-related KAP. In the knowledge section, respondents were asked about the degree to which they understood the difference between NIPT and MSS, and the source from which they obtained NIPT-related information. In the attitude section, the preference for pregnant women was investigated. The practices section explored whether respondents had taken the NIPT, and received pretest counseling and posttest explanation for the results.

The last part assessed the satisfaction among women who had taken NIPT using a 5-point Likert scale (1 = very dissatisfied to 5 = very satisfied). The scale had 7 domains, including the charge, received information, service of the doctor, service of the nurse, hospital environment, waiting time, and results explanation. The internal consistency reliability of the satisfaction scale was high (Cronbach’s Alpha = 0.995) [27]. An exploratory factor analysis was applied to establish the dimensions of the satisfaction scale. Items with factor loadings larger than 0.4 and cross-loadings lower than 0.3 would be kept [28]. The Kaiser–Meyer–Olkin sampling adequacy measure was 0.929, and the results of Bartlett sphericity were statistically significant (χ^2^ = 7724.256, *p* < 0.001), which indicated that the factor analysis was appropriate. One factor with eigenvalues greater than one was extracted by principal component analysis, and it accounted for 75.70% of the total variance. The loadings of all items were larger than 0.4 (Table 1), so all items were retained in the analysis.

### 2.4. Data Analysis

EpiData 3.1 (The EpiData Association, Odense, Denmark) and IBM Statistical Package for the Social Sciences (SPSS) 20.0 (IBM, Armonk, NY, USA) were used for data entry and analysis respectively. Descriptive analyses were presented to describe the socio-demographic characteristics of participants, and their NIPT-related KAP. Meanwhile, the chi-square test and Wilcoxon rank sum test were used to compare the differences between Shenzhen and Zhengzhou groups. As the background information of pregnant women might be quite different between Shenzhen and Zhengzhou, propensity score matching (PSM) was applied based on the multivariable logistic regression. Participants from Shenzhen or Zhengzhou were matched by 1:1 within the PS score of 0.02, and covariates were socio-demographic characteristics. After matching, differences in the satisfaction level towards NIPT between Shenzhen and Zhengzhou groups were analyzed using the paired Wilcoxon rank sum test.

Multivariable logistic regression models were applied to identify factors associated with acceptability and satisfaction level towards NIPT. In the first model, the dependent variable was whether the participant had received NIPT. The independent variables included the socio-demographic characteristics of women, their knowledge of NIPT, and the survey site. In the second model, the dependent variable was the overall satisfaction level, and the independent variables included “whether received pretest counseling/posttest results explanation”, along with the variables used in the first model. The overall satisfaction level was defined as the average score of seven domains’ satisfaction scores and it was divided into a dichotomous variable (<3 = dissatisfied, 3–5 = satisfied). Results were considered statistically significant if *p* < 0.05. Forward likelihood ratio method was used to select variables, and only variables with *p* < 0.05 were included in the final regressions.

## 3. Results

### 3.1. Sample Characteristics

A total of 622 and 1201 pregnant women from Shenzhen and Zhengzhou completed the questionnaire. In terms of socio-demographic characteristics, there were statistically significant differences between the two groups, except for the parity. Compared with women from Zhengzhou, women from Shenzhen tended to be older (≥35 years old), live in urban areas, have a higher educational level and annual family income, along with smaller family sizes. The majority of participants from Shenzhen (60.77%) had Basic Medical Insurance for Urban Employees, while Basic Medical Insurance for Urban and Rural Residents dominated in Zhengzhou participants (67.03%). The detailed socio-demographic characteristics of participants are summarized in Table 2.

### 3.2. The Knowledge and Attitude of NIPT

In all, 543 (87.30%) Shenzhen women and 769 (64.03%) Zhengzhou women had heard of NIPT, but participants from Shenzhen had better knowledge (*p* < 0.001). The main approaches for women to obtain NIPT-related information included doctors, relatives, friends, and colleagues, and websites. The majority of participants knew which hospital can provide NIPT for them, and the proportion was higher in Shenzhen (Shenzhen vs. Zhengzhou: 67.40% vs. 61.12%, *p* < 0.001). Meanwhile, the proportion of participants who had been aware of the difference between NIPT and MSS was higher in Shenzhen—70.72% of them showed good awareness, while the proportion in Zhengzhou was only 61.50% (*p* < 0.001). Details of pregnant women’s knowledge of NIPT are presented in Table 3.

To explore the women’s attitude towards NIPT, we invited them to choose an ideal screening strategy after a detailed explanation of the major characteristics of both NIPT and MSS. As a result, 40.84% and 50.96% of Shenzhen participants chose “only NIPT” and “Sequential screening of MSS and NIPT” respectively, while the percentages were 22.48% and 57.95% in Zhengzhou participants. The rest of the participants chose “Only MSS” or did not have a clear preference. It presented that, compared with Zhengzhou, participants in Shenzhen had a greater willingness to receive NIPT (*p* < 0.001).

### 3.3. The Practices of NIPT

In all, 517 (83.12%) Shenzhen women and 655 (54.54%) Zhengzhou women had taken NIPT to screen for fetus aneuploidy, and the acceptance rates were much higher in the former group (*p* < 0.001). Several factors were associated with NIPT acceptability based on logistic regression. Respondents from Shenzhen were more likely to accept NIPT than those from Zhengzhou (odds ratio (OR): 3.85, 95% confidence interval (CI): 2.71–5.46, *p* < 0.001). Women who participated in Basic Medical Insurance for Urban Employees had a higher odd of acceptance than those who did not (OR:1.55, 95% CI: 1.15–2.09, *p* = 0.004). Besides, women with higher annual household income and awareness of NIPT were more likely to take NIPT (*p* < 0.05) (Table 4).

For women who had received NIPT, their reasons for taking the test varied. The most common reasons for Shenzhen participants were: no risk to the fetus (57.25%), high accuracy (54.93%), the recommendation from doctors (39.85%), and suitable for longer gestation (22.44%). In contrast, the most common reasons in Zhengzhou participants were: the recommendation from doctors (74.35%), no risk to the fetus (73.59%), and high accuracy (47.63%). Comparatively, Zhengzhou participants relied more heavily on doctor’s advice and Shenzhen participants attached more importance to the advantage of NIPT (Table 5).

More than 50% of participants considered that inadequate publicity was the major problem in current NIPT practice. About 40% of participants were dissatisfied with the limited aneuploid disorders that NIPT can detect. Moreover, 52.82% of Zhengzhou participants thought that NIPT was too expensive, while far fewer Shenzhen participants (15.28%) agreed. In terms of counseling, the majority of respondents consulted the doctor before and after NIPT about test-related information and results explanation, and the counseling rates were higher among Zhengzhou participants (*p* < 0.001) (Table 5).

### 3.4. The Satisfaction Towards NIPT

About 98% of pregnant women were satisfied with the clinical practice of NIPT, except for the charge domain, which 5.07 % and 17.05% of Shenzhen and Zhengzhou participants were dissatisfied with. PSM analysis was used to match the Shenzhen and Zhengzhou participants to control the socio-demographic heterogeneity between the two groups. In all, 292 pairs of participants were matched. The results presented that there was no significant difference in the satisfaction of the charge of NIPT between the two groups (*p* = 0.60). However, in other domains, the satisfaction levels were significantly higher among Zhengzhou participants compared with those from Shenzhen (*p* < 0.001) (Table 6).

To discover factors that influenced the overall satisfaction in NIPT, multivariable logistic regression was applied. The overall satisfaction level was lower among Shenzhen participants compared with Zhengzhou participants (OR: 0.07, 95% CI: 0.01–0.58, *p* = 0.013). Women who had given birth before were more satisfied with NIPT than those who had not (OR: 5.78, 95% CI: 1.52–22.02, *p* = 0.010). Women who had received test results explanation from doctors held a more positive attitude towards NIPT (OR: 12.21, 95%CI: 2.94–50.73, *p* = 0.001). Furthermore, participants with higher annual household income tended to be satisfied with NIPT (*p* = 0.030) (Table 7).

## 4. Discussion

In all, about 72% of participants in the two cities had heard of NIPT, and 84% of them were willing to receive it. About 64% of participants had taken NIPT, and the majority of them felt satisfied with its service. A study conducted in Hong Kong also discovered that approximately 70% of Chinese women had a certain degree of knowledge in NIPT, and 82% of them tended to choose NIPT as the primary screening test or the one used after an MSS positive result [29]. These were in line with our results. Besides, as the socio-demographic characteristics differed significantly between the two groups, the study used multivariable regression models to compare the response of pregnant women from Shenzhen and Zhengzhou, which respectively represent the “insurance coverage” and “out-of-pocket” payment schemes. The results presented that payment schemes could significantly affect women’s acceptability of and satisfaction with NIPT.

### 4.1. Knowledge and Affordability Can Affect Women’s Acceptability

The acceptance rate of NIPT was much higher among Shenzhen participants. On the one hand, it might relate to the fact that, compared with the Zhengzhou group, a higher proportion of Shenzhen participants knew NIPT. Correspondingly, Shenzhen participants tended to choose NIPT based on their understanding of the test, not barely on doctor’s advice. Besides, after we introduced the characteristics of NIPT and MSS to pregnant women, an extra 25% of Zhengzhou participants expressed that they would have chosen NIPT if they had known the information earlier. This demonstrated the importance of NIPT-related knowledge on women’s attitude. On the other hand, because of the high price of NIPT and “out-of-pocket” payment scheme, the accessibility of NIPT was still low in Zhengzhou. Even for women who had taken NIPT, more than half of them considered that it was too expensive. The finding is consistent with the work of Allyse et al., who performed an open-question survey in the United States. The work discovered that a better understanding of the accuracy of NIPT could motivate the choice of NIPT, while the cost was an important consideration to justify the selection of MSS over NIPT [30]. The finding also accords with the results from a Switzerland hospital, which found that the uptake of NIPT increased notably after nationwide health insurance coverage for NIPT [31].

### 4.2. Payment Pattern May Affect Women’s Satisfaction

In terms of satisfaction level, the majority of pregnant women were satisfied with the NIPT service. Comparatively, Zhengzhou participants gave a higher level of praise on service delivery and information explanation domains than those in Shenzhen. A possible explanation for this might be that more Zhengzhou participants consulted the doctor for test-related information and asked for results explanation. Besides, the “out-of-pocket” payment scheme might force pregnant women in Zhengzhou to think seriously before making the choice. They would choose to receive NIPT only if they considered that the value of high accuracy brought by NIPT outweighed its price. This may also contribute to their greater satisfaction with NIPT. On the contrary, two groups showed a similar satisfaction level in the charge domain. This finding suggests that, for women who choose NIPT, the cost is not their major concern in satisfaction evaluation. Therefore, to enhance satisfaction, more attention should be paid to the improvement of service delivery and the hospital environment.

### 4.3. Genetic Counseling Help to Improve Women’s Satisfaction

Except for the payment schemes, genetic counseling could also affect pregnant women’s overall satisfaction level of NIPT. The result of logistic regression indicated that receiving genetic counseling could positively enhance women’s satisfaction. This finding supported the recommendation to increase the availability of genetic counseling, which was also stated by ISPD, NSGC, and ACMG. In accordance with the present results, previous studies have also demonstrated that pregnant women gave high praise on pretest and posttest genetic counseling, as they provide enough information and help in the decision-making process [32,33]. Pretest counseling should tell women about the benefits and disadvantages of NIPT, including the scope of detectable aneuploid disorders, its accuracy, and limitation [15]. This information can support women in their decision-making process. Posttest counseling aims to give a comprehensive explanation of the NIPT results. For women with a high-risk result, the genetic counselors should emphasize that NIPT is not diagnostic and it has the possibility of being false-positive, so invasive prenatal diagnosis is recommended to confirm the disease [14]. Women with a “screen-negative” result need to know the existence of false-negative results. The National Health Commission of China requests that genetic counseling should be provided for every pregnant woman with a high-risk result. However, genetic counseling has not been recognized as a formal profession in China, and professional genetic counseling services were only available in a few large cities [34]. There is a huge gap between the need and the supply.

### 4.4. Recommendations

In conclusion, three problems existed in China’s NIPT practice, including inadequate publicity, low affordability under the “out-of-pocket” payment scheme, and a lack of genetic counseling. Three recommendations are given to improve Chinese pregnant women’s accessibility of and satisfaction with NIPT. First, the internet has been widely used in the health education program [35,36]. Authorities and hospitals can consider providing professional information on prenatal screening and aneuploid disorders online, such as creating official websites or apps. This could be an effective and efficient way to boost pregnant women’s knowledge. Second, it is recommended that provincial bureaus should provide a certain degree of subsidies for NIPT or cover it in social medical insurance. Third, greater efforts are needed to promote the development of genetic counseling in China. Besides, based on the literature finding, the problems existing in China were also presented in many other countries. Therefore, further research can make efforts to summarize the successful experience on these topics around the world.

### 4.5. Limitations

One limitation of this study is that due to convenience sampling, participants in our study may be unrepresentative. So, the results should be interpreted with caution. Besides, due to the statistically significant differences between the Shenzhen and Zhengzhou groups in socio-demographic characteristics, it is difficult to compare the two groups objectively. Although we tried to control the confounders by PSM and logistic regression, there could be some important factors that we did not measure and put in the model, and this could introduce bias. Furthermore, there is a great distance between Shenzhen and Zhengzhou, which leads to some cultural and social differences between the two cities. These differences may also influence residents’ KAP and satisfaction towards prenatal screening and aneuploid disorders. Further studies need to be carried out to compare the NIPT-related KAP and the satisfaction of individuals with similar cultural and social backgrounds.

## 5. Conclusions

Knowledge and affordability could significantly affect the acceptability of NIPT, while genetic counseling could improve women’s satisfaction with NIPT. Therefore, strengthening related health education, providing certain degrees of subsidies on NIPT, and promoting the development of genetic counseling could be effective approaches to enhance the accessibility and satisfaction of NIPT in China.

## Figures and Tables

**Table 1:** The loading of each item based on exploratory factor analysis of the satisfaction scale.

Items	Factor 1
Charge	0.658
Received information	0.875
Service of doctor	0.917
Service of nurse	0.902
Hospital environment	0.915
Waiting time	0.889
Results explanation	0.904
% of variance	75.697

**Table 2:** The detailed socio-demographic characteristics of participants (*n* (%)) (USD 1 = CNY 6.8985).

Participant Characteristics	Shenzhen (*n* = 622)	Zhengzhou (*n* = 1201)	χ^2^/Z	*p*	Participant Characteristics	Shenzhen (*n* = 622)	Zhengzhou (*n* = 1201)	χ^2^/Z	*p*
Age *					Occupation				
≤24	28 (4.50)	153 (12.74)	−6.26	<0.001	A	52 (8.36)	47 (3.91)	181.17	<0.001
25–34	429 (68.97)	839 (69.86)			B	99 (15.92)	275 (22.90)		
≥35	162 (26.05)	209 (17.40)			C	81 (13.02)	108 (8.99)		
Missing	3 (0.48)	0 (0.00)			D	52 (8.36)	120 (9.99)		
Educational level *					E	1 (0.16)	25 (2.08)		
Middle school or below	32 (5.14)	260 (21.65)	−13.37	<0.001	F	21 (3.38)	41 (3.41)		
High school	88 (14.15)	297 (24.73)			Unemployed	78 (12.54)	390 (32.47)		
Three-year college	189 (30.39)	362 (30.14)			Others	222 (35.69)	190 (15.82)		
Bachelor or above	300 (48.23)	269 (22.40)			Missing	16 (2.57)	5 (0.42)		
Missing	13 (2.09)	13 (1.08)			Medical insurance (Multiple answers) **				
Annual household income (thousand USD) *					UE	378 (60.77)	345 (28.73)	195.83	<0.001
<10	80 (12.86)	367 (30.56)	−16.89	<0.001	URR	199 (31.99)	805 (67.03)	201.15	<0.001
10–14	69 (11.09)	410 (34.14)			CHI	32 (5.14)	69 (5.75)	0.16	0.686
14–28	209 (33.60)	309 (25.73)			Missing	41 (6.59)	49 (4.08)		
≥28	243 (39.07)	101 (8.41)			Parity				
Missing	21 (3.38)	14 (1.17)			Nulliparous	275 (44.21)	526 (43.80)	2.18	0.140
Number of family members *					Multiparous	301(48.39)	669 (55.70)		
≤3	317 (50.96)	523 (43.55)	−3.32	0.001	Missing	46 (7.40)	6 (0.50)		
4–6	274 (44.05)	667 (55.54)			Residence				
≥7	12 (1.93)	11 (0.92)			Urban	527 (84.73)	728 (60.62)	133.03	<0.001
Missing	19 (3.05)	0 (0.00)			Rural	65 (10.45)	432 (35.97)		
					Missing	30 (4.82)	41 (3.41)		

A—Managers; B—Professional and technicians; C—Clerical support workers; D—Service and sales workers; E—Skilled agricultural, forestry, and fishery workers; F—Plant and machine operators, and assemblers; UE—Basic Medical Insurance for Urban Employees; URR—Basic Medical Insurance for Urban and Rural Residents; CHI—Commercial Health Insurance. * Wilcoxon rank sum test used. ** Each answer was treated as a dichotomous variable and the chi-square test was conducted separately.

**Table 3:** The pregnant women’s knowledge of non-invasive prenatal testing (*n* (%)).

Questions	Shenzhen (*n* = 543)	Zhengzhou (*n* = 769)	χ^2^/Z	*p*
Main approaches to obtain NIPT-related information (Multiple answers) **				
Lectures	42 (7.73)	60 (7.80)	0.00	0.964
Doctors	393 (72.38)	511 (66.45)	5.22	0.022
Relatives, friends and colleagues	130 (23.94)	258 (33.55)	14.11	<0.001
Websites	71 (13.08)	100 (13.00)	0.00	0.970
Newspaper or TV	27 (4.97)	21 (2.73)	4.54	0.033
Others	14 (2.58)	1 (0.13)	16.88	<0.001
Missing	0 (0.00)	0 (0.00)		
Do you know where you can receive NIPT?				
Yes	366 (67.40)	470 (61.12)	267.36	<0.001
No	176 (32.41)	294 (38.23)		
Missing	1 (0.18)	5 (0.65)		
Are you aware of the difference between NIPT and MSS? *				
Completely aware	35 (6.45)	19 (2.47)	−5.64	<0.001
Very aware	121 (22.28)	91 (11.83)		
Somewhat aware	228 (41.99)	363 (47.20)		
Not so aware	147 (27.07)	263 (34.20)		
Not at all aware	13 (2.39)	32 (4.16)		
Missing	0 (0.00)	1 (0.13)		

NIPT—non-invasive prenatal testing; MSS—maternal serum screening. * Wilcoxon rank sum test used. ** Each answer was treated as a dichotomous variable and the chi-square test was conducted separately.

**Table 4:** Logistic regression analysis of factors associated with acceptability towards non-invasive prenatal testing (USD 1 = CNY 6.8985).

Variables		B	OR (95% CI)	*p*
Research Location	Zhengzhou		1	<0.001
	Shenzhen	1.35	3.85 (2.71, 5.46)	
UE	Not participant		1	0.004
	Participant	0.44	1.55 (1.15, 2.09)	
Annual household income (thousand USD)	<10		1	0.009
	10–14	0.09	1.09 (0.74, 1.60)	
	14–28	0.51	1.66 (1.11, 2.48)	
	≥28	0.66	1.93 (1.19, 3.12)	
Awareness	Not at all aware		1	<0.001
	Not so aware	0.09	1.09 (0.54, 2.20)	
	Somewhat aware	0.69	1.99 (0.99, 4.00)	
	Very aware	1.13	3.09 (1.37, 6.96)	
	Completely aware	0.63	1.88 (0.69, 5.12)	
Constant		−0.63		

UE—Basic Medical Insurance for Urban Employees; B—coefficient; OR—odds ratio; CI—confidence interval; Awareness—awareness of the difference between non-invasive prenatal testing and maternal serum screening. Note: Forward Likelihood Ratio method was used to select variables and only variables with *p* < 0.05 were included in the final regression.

**Table 5:** The practices of non-invasive prenatal testing among pregnant women (*n* (%)).

Questions	Shenzhen (*n* = 517)	Zhengzhou (*n* = 655)	χ^2^	*p*
Reasons for accepting NIPT (Multiple answers) **				
No risk to the fetus	296 (57.25)	482 (73.59)	35.51	<0.001
High accuracy	284 (54.93)	312 (47.63)	5.86	0.015
Suitable for longer gestation	116 (22.44)	74 (11.30)	26.12	<0.001
Insurance covered	16 (3.09)	67 (10.23)	22.49	<0.001
Shorter waiting time	27 (5.22)	75 (11.45)	14.24	<0.001
Recommendation from doctors	206 (39.85)	487 (74.35)	143.99	<0.001
Others	7 (1.35)	30 (4.58)	9.90	0.002
Missing	0 (0.00)	1 (0.15)		
The perceived problems of NIPT practice (Multiple answers) **				
Too expensive	79 (15.28)	346 (52.82)	174.23	<0.001
Inadequate publicity	281 (54.35)	367 (56.03)	0.16	0.691
Distrust it	4 (0.77)	4 (0.61)	0.12	0.726
Only detect few aneuploid disorders	186 (35.98)	263 (40.15)	1.79	0.181
Others	72 (13.93)	11 (1.68)	66.71	<0.001
Missing	8 (1.55)	4 (0.61)		
Did you consult the doctor before accepting NIPT?				
Yes	454 (87.81)	639 (97.56)	196.25	<0.001
No	57 (11.03)	11 (1.68)		
Missing	6 (1.16)	5 (0.76)		
Did you consult the doctor for NIPT results explanation?				
Yes	481 (93.04)	621 (94.81)	289.78	<0.001
No	28 (5.42)	19 (2.90)		
Missing	8 (1.55)	15 (2.29)		

NIPT—non-invasive prenatal testing. ** Each answer was treated as a dichotomous variable and the chi-square test was conducted separately.

**Table 6:** The satisfaction towards non-invasive prenatal testing among matched pairs of pregnant women (*n* (%)).

	Charge	Received Information	Service of Doctor	Service of Nurse	Hospital Environment	Waiting Time	Results Explanation
Shenzhen							
Completely dissatisfied	2 (0.68)	1 (0.34)	0 (0.00)	0 (0.00)	0 (0.00)	0 (0.00)	0 (0.00)
Very dissatisfied	14 (4.79)	6 (2.05)	2 (0.68)	2 (0.68)	2 (0.68)	0 (0.00)	2 (0.68)
Somewhat satisfied	129 (44.18)	106 (36.30)	94 (32.19)	88 (30.14)	96 (32.88)	99 (33.90)	93 (31.85)
Very satisfied	80 (27.40)	103 (35.27)	95 (32.53)	99 (33.90)	98 (33.56)	102 (34.93)	103 (35.27)
Completely satisfied	67 (22.95)	76 (26.03)	101 (34.59)	103 (35.27)	96 (32.88)	91 (31.16)	94 (32.19)
Mean rank	3.67	3.85	4.01	4.04	3.99	3.97	3.99
Zhengzhou							
Completely dissatisfied	0 (0.00)	0 (0.00)	0 (0.00)	0 (0.00)	0 (0.00)	0 (0.00)	0 (0.00)
Very dissatisfied	30 (10.27)	0 (0.00)	0 (0.00)	0 (0.00)	0 (0.00)	0 (0.00)	0 (0.00)
Somewhat satisfied	90 (30.82)	19 (6.51)	15 (5.14)	9 (3.08)	35 (11.99)	31 (10.62)	19 (6.51)
Very satisfied	108 (36.99)	174 (59.59)	175 (59.93)	169 (57.88)	159 (54.45)	165 (56.51)	169 (57.88)
Completely satisfied	64 (21.92)	99 (33.90)	102 (34.93)	114 (39.04)	98 (33.56)	96 (32.88)	104 (35.62)
Mean rank	3.71	4.28	4.3	4.36	4.22	4.22	4.29
*p*	0.60	<0.001	<0.001	<0.001	<0.001	<0.001	<0.001

**Table 7:** Logistic regression analysis of factors associated with the overall satisfaction level towards non-invasive prenatal testing (USD 1 = CNY 6.8985).

Variables		B	OR (95% CI)	*p*
Research Location	Zhengzhou		1	0.013
	Shenzhen	−2.64	0.07 (0.01, 0.58)	
Parity	Nulliparous		1	0.010
	Multiparous	1.75	5.78 (1.52, 22.02)	
Annual household income (thousand USD)	<10		1	0.030
	10–14	2.17	8.75 (0.89, 86.06)	
	14–28	2.05	7.77 (1.62, 37.18)	
	≥28	1.77	5.87 (1.37, 25.21)	
Test results explanation received	No		1	0.001
	Yes	2.50	12.21 (2.94, 50.73)	
Constant		1.68		

B—coefficient; OR—odds ratio; CI—confidence interval. Note: Forward Likelihood Ratio method was used to select variables and only variables with *p* < 0.05 were included in the final regression.
